# Analysis of significance of CARD11 and MYO1G expressions in pulmonary tuberculosis and their predictive value for prognosis of recurrence

**DOI:** 10.5937/jomb0-54554

**Published:** 2025-03-21

**Authors:** Fengxia Liu, Shengxun Lin, Li Li, Bei Xu, Tingting Chang

**Affiliations:** 1 Shandong University, Shandong Public Health Clinical Center, Department of Tuberculosis 7 Ward, Jinan, China; 2 Nantong Hospital of Shanghai University, The Sixth People's Hospital of Nantong, Nantong, Medical Laboratory, Nantong, China; 3 Feicheng People's Hospital, Department of Respiratory and Critical Care Medicine, Lung function room, Feicheng, China; 4 Jinan Fourth People's Hospital, Department of Infectious Diseases, Jinan, China

**Keywords:** pulmonary tuberculosis, CARD11, MYO1G, T cell activation, analysis of biological information, tuberkuloza pluća, CARD11, MYO1G, aktivacija T-ćelija, analiza bioloških informacija

## Abstract

Pulmonary tuberculosis (PTB) is one of the most common infectious diseases worldwide, with extremely high morbidity and mortality. An in-depth understanding of the molecular pathogenesis of PTB is crucial for finding novel diagnostic and therapeutic approaches in the future. In this study, we identified 52 differentially expressed genes (DEGs) from the GSE34608 dataset in the Gene Expression Omnibus (GEO) database, and the protein-protein interaction (PPI) network showed that these DEGs included 42 nodes and 20 edges. We found that CARD11 and MYO1G were closely associated with T cell activation by functional enrichment analysis. Upon further clinical case analysis, we found that the expressions of CARD11 and MYO1G in the peripheral blood of patients with PTB were lower than those of healthy individuals. Meanwhile, through Receiver operating characteristic (ROC) curve analysis, we found that CARD11 and MYO1G showed excellent diagnostic effects on the occurrence and prognosis of PTB recurrence. Thus, CARD11 and MYO1G are promising indicators for assessing PTB in the future.

## Abstract (sr)

Tuberkuloza pluća (PTB) je jedna od najčešćih zaraznih bolesti u svetu, sa izuzetno visokim stopama morbiditeta i mortaliteta. Dublje razumevanje molekularne patogeneze PTB je ključno za pronalaženje novih dijagnostičkih i terapijskih pristupa u budućnosti. U ovoj studiji smo identifikovali 52 različito eksprimirana gena (DEG) iz skupa podataka GSE34608 u bazi Gene Expression Omnibus (GEO), a mreža interakcija protein-protein (PPI) je pokazala da ovi DEG uključuju 42 čvora i 20 ivica. Utvrdili smo da su CARD11 i MYO1G blisko povezani sa aktivacijom T-ćelija putem analize funkcionalnog obogaćivanja. Daljom analizom kliničkih slučajeva, utvrdili smo da su ekspresije CARD11 i MYO1G u perifernoj krvi pacijenata sa PTB bile niže u odnosu na zdrave pojedince. Takođe, analizom ROC krive smo utvrdili da CARD11 i MYO1G pokazuju izuzetne dijagnostičke efekte u pogledu pojave i prognoze recidiva PTB. Stoga su CARD11 i MYO1G indikatori za procenu PTB koji obećavaju.

## Introduction

Pulmonary tuberculosis (PTB), an infectious respiratory disease caused by Mycobacterium tuberculosis, is one of the most common infectious diseases worldwide [1]. According to the World Health Organization, in 2017 over 1.7 billion people were latently infected with PTB globally, and 10 million new PTB cases each year [2]. Among them, about 1.3 million patients eventually died of PTB, with an average mortality rate of up to 17 per 100,000 [3]. PTB affects people of all ages, placing significant pressure on the modern healthcare system [4]. There is a lack of effective PTB prevention measures due to the limitations of PTB diagnostic schemes and difficulties in achieving widespread clinical screening [5]. An in-depth understanding of the pathogenesis of PTB is therefore crucial for finding novel diagnostic and therapeutic approaches in the future.

The transcriptome technologies based on gene microarrays or high-throughput sequencing platforms have been widely used in biomarker screening for human diseases. With the application of gene microarrays and second-generation sequencing, vast amounts of biological data have been generated, most of which have been uploaded to public databases; integration and reanalysis of these data will provide valuable insights for our research [6] [7]. Some studies have also analysed PTB’s differentially expressed genes (DEGs) through online databases and proposed new research directions. For example, the study of Sun Y et al. [8] found that CCL20, PTGS2, ICAM1, TIMP1, MMP9, CXCL8, and IL6 are closely related to the occurrence of PTB by analysing the biological information and have the potential to be used as biomarkers. Li Y et al. [9] established a model for distinguishing latent PTB from active PTB using DEGs in PBT. However, further supplementation and refinement are still needed to achieve clinical application.

In the present study, we were to download PTB-related data from Gene Expression Omnibus (GEO), screen the DEGs between PTB patients and healthy controls, and perform functional pattern analysis to identify PTB-related biomarkers, providing a foundation for elucidating the molecular mechanism of PTB occurrence and developing targeted therapies.

## Materials and methods

### Data source

The target dataset was GSE34608 (Gene and microRNA expression in pulmonary tuberculosis and sarcoidosis). It contained 43 whole blood samples from patients with PTB and 28 whole blood samples from healthy individuals. The GPL6480 (Agilent-014850 Whole Human Genome Microarray 4×44K G4112F) platform was used for data analysis.

### DEGs analysis

The results of the above dataset were imported into the GEO2R online tool for DEG screening. After removing duplicate and unnamed genes, |LogFC| >3 and P<0.05 were used as the screening criteria. Based on the filtered DEGs, an expression heatmap was plotted using the Series Matrix File.

### Protein-protein interaction network analysis

The filtered DEGs were uploaded to the STRING database (https://cn.string-db.org/) to construct a protein-protein interaction (PPI) network and then visualised using the Cytoscape software with a composite score of >0.4 as the significance threshold. The degree values of the core genes in the PPI network were calculated using default parameters, and the clustering module analysis of the PPI network was performed using the MCODE plug-in.

### Functional enrichment analysis

GO and KEGG enrichment analyses were performed on the co-differentially expressed genes using the online database DAVID (https://david.ncif-crf.gov/home.jsp), in which GO analysis included cell component (CC), biological process (BP), and molecular function (MF).

### Clinical cases

Fifty-two patients with PTB were admitted to our hospital from March 2022 to June 2023, and 58 healthy subjects undergoing physical examinations during the same period were enrolled as the study objects. Patients with PTB were assigned to the research group, and healthy subjects to the control group. The study has been approved by the Ethics Committee of Shandong Public Health Clinical Center (No.GWLCZXEC2023-75-1). All subjects signed the informed consent form.

### Inclusion and exclusion criteria

Inclusion criteria: (1) Subjects aged 18–70 years. (2) Subjects in the research group were supposed to meet the diagnostic guidelines for PTB and be confirmed by chest CT or X-ray, sputum smear culture and Mycobacterium tuberculosis culture, etc. (3) Subjects in the control group showed normal physical examination results and reported no apparent discomfort. Exclusion Criteria: (1) Subjects with a history of tuberculosis or those with drug-resistant Myco bacterium tuberculosis. (2) Those who had undergone PTB-related treatment before enrollment. (3) Those with autoimmune diseases or cardiovascular and cerebrovascular diseases. (4) Those with malignant tumours. (5) Those who were in pregnancy or breastfeeding period. (6) Those with other pulmonary diseases.

### Methods

Fasting peripheral blood was obtained from subjects in both groups at admission, centrifuged for serum separation, and subjected to total RNA extraction with the Trizol kit. Then, 30 μg of the total RNA was reversely transcribed into cDNA for PCR reaction. 2-ΔΔCT calculated the relative expression of CARD11 and MYO1G with GAPDH as the internal reference. The primer sequences were designed and constructed by GenScript Biotech Corporation (Nanjing), as contracted (Table 1).

**Table 1:** Comparison of the expression of SHOX2, RASSF1A, and PTGER4 in cancer tissues and paracancerous tissues of LC patients (n (%)).

	F (3’-5’)	R (3’-5’)
CARD11	CGCACTTCCTGATGAACGAG	GTCCCGCTCTTCCTTCATCT
MYO1G	GGGTAGAAGGTTATTCGTTGTGTATTTC	CAATATACACAAAATACTTAACTCACGTCCT
GAPDH	GGGAAACTGTGGCGTGAT	GAGTGGGTGTCGCTGTTGA

### Prognosis follow-up

Patients in the research group were followed up for 6 months (regular review), with an interval between two consecutive visits not more than 1 month, and the prognosis of PTB recurrence (recurrence was defined as the presence of any PTB active symptoms) was recorded.

### Statistical analysis

Statistical analysis was performed with the statistical software SPSS24.0. The chi-square test compared enumeration data [n (%)]. Measurement data (x̄ ± s) were compared with the independent sample t-test. The receiver operator characteristic (ROC) curve analysed the diagnostic value. P<0.05 was considered statistically significant.

## Results

### Results of DEG screening

**Figure 1:**
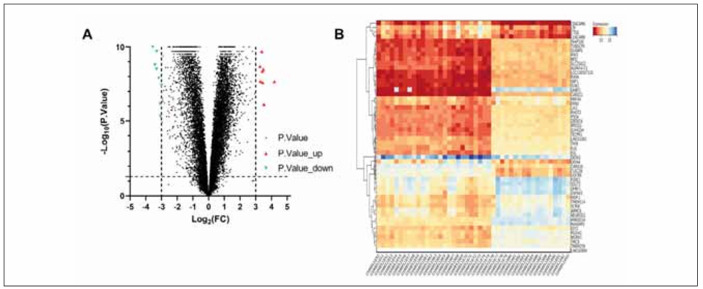
Results of DEG screening.<br>A: volcano plot of screened DEGs. B: heatmap of DEGs expression.

After the removal of duplicate and unnamed genes, the GSE34608 dataset contained a total of 22773 genes. Through GEO2R analysis and screening, 52 DEGs were identified, of which 9 were upregulated and 43 were downregulated (Figure 1A). Subsequently, we mapped the expression heatmap of these DEGs (Figure 1B). We could see that EVL and DEFA4 were the most significantly differentially expressed DEGs in the sample of 43 PTB patients.

### PPI analysis results

**Figure 2:**
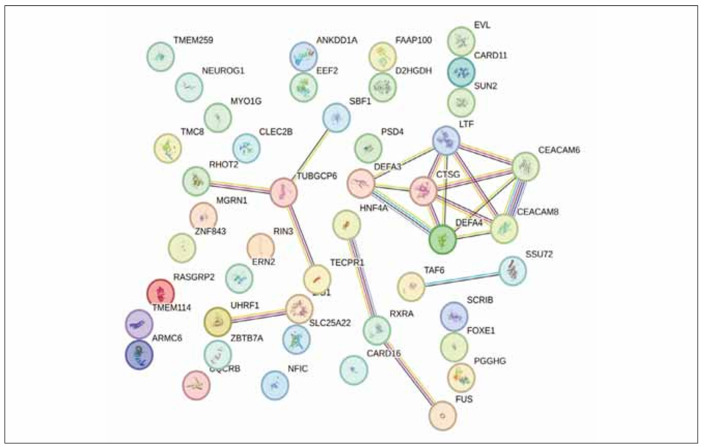
PPI analysis results.

The PPI network of the DEGs consists of 42 nodes and 20 edges, including 2 core genes of TUBGCP6 and CTSG (Figure 2).

### Functional enrichment analysis results

**Figure 3:**
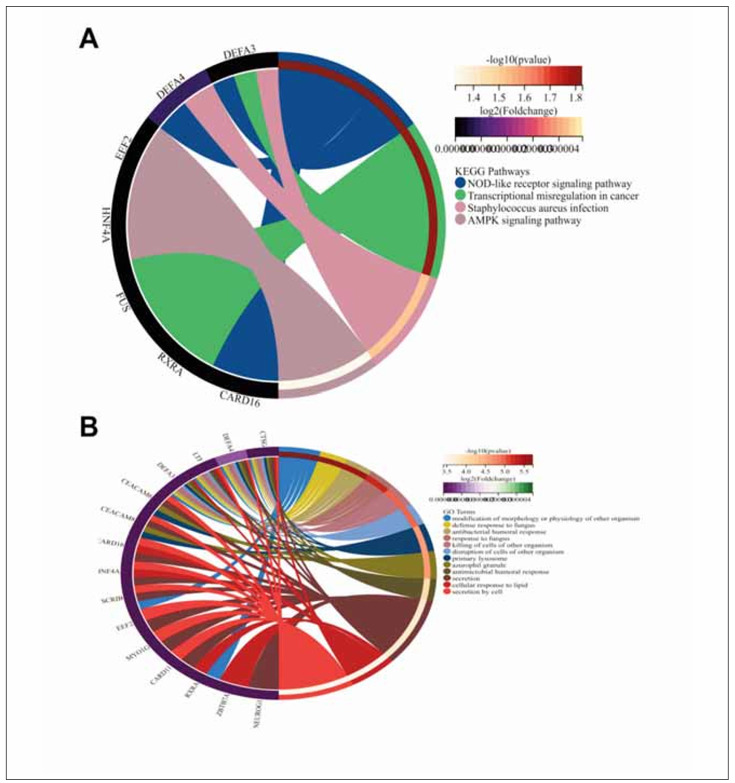
Functional enrichment analysis results<br>A: Results of KEGG analysis. B: Results of GO analysis.

The results of KEGG and GO analysis showed that keywords strongly associated with these DEGs included »modification of morphology or physiology of another organism« and »NOD-like receptor signalling pathway« (Figure 3). In GO analysis, we screened all the analysed results about T cell function, in which it is seen that CARD11 involves activation, costimulation, differentiation, differentiation in the thymus, proliferation of T cells, receptor signalling pathway, and selection, while MYO1G involves mediated immunity, migration of T cells (detailed in Table 2).

**Table 2:** DEGs associated with T cell activation.

Description	GeneRatio	BaRatio	p-value	p.adjust	g-value	Count	Gene ID
T cell activation	1/42	443/17910	0.651161427	0.715878628	0.587914011	1	CARD11
Tcell costimulation	1/42	55/17910	0.121311642	0.320651884	0.263334772	1	CARD11
T cell differentiation	1/42	234/17910	0.424776958	0.556378579	0.456924888	1	CARD11
T cell differentiation<br>in thymus	1/42	69/17910	0.149820482	0.355606061	0.292040825	1	CARD11
T cell mediated immunity	1/42	88/17910	0.187071039	0.389831906	0.320148738	1	MYO1G
T cell migration	1/42	59/17910	0.129550897	0.328969653	0.270165725	1	MYO1G
T cell proliferation	1/42	177/17910	0.341387956	0.497600676	0.408653643	1	CARD11
T cell receptor signalling<br>pathway	1/42	180/17910	0.346056844	0.49900161	0.409804157	1	CARD11
T cell selection	1/42	45/17910	0.100378998	0.296908574	0.243835622	1	CARD11

### Clinical expression analysis

**Figure 4:**
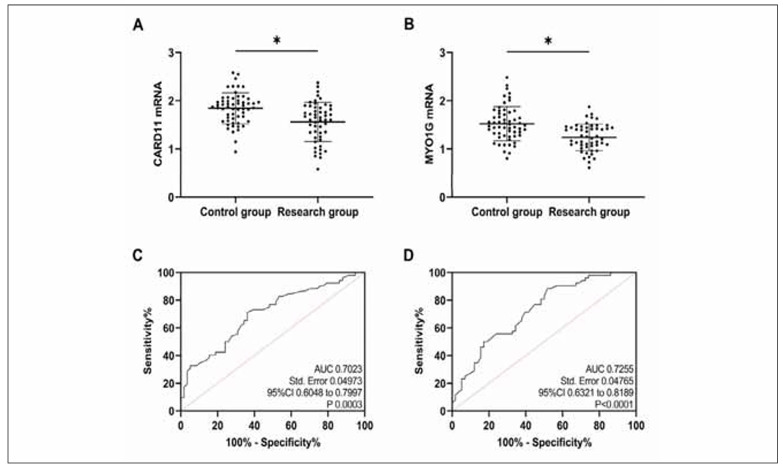
Clinical expression analysis.<br>A: Comparison of CARD11. B: Comparison of MYO1G. C: ROC curves for diagnosis of PTB by CARD11. D: ROC curves for diagnosis of PTB by MYO1G. *P<0.05.

Compared with the control group, CARD11 and MYO1G mRNA expression were reduced in the research group (P<0.05, Figure 4A and Figure 4B). As indicated by the ROC curves, when CARD11 mRNA was <1.80, its sensitivity and specificity in diagnosing PTB occurrence were 71.15% and 63.79%, respectively (P<0.05, Figure 4C); when MYO1G mRNA was <1.50, its sensitivity and specificity in diagnosing PTB occurrence were 88.46% and 48.28%, respectively (P<0.05, Figure 4D).

### Assessment of prognosis of recurrence

**Figure 5:**
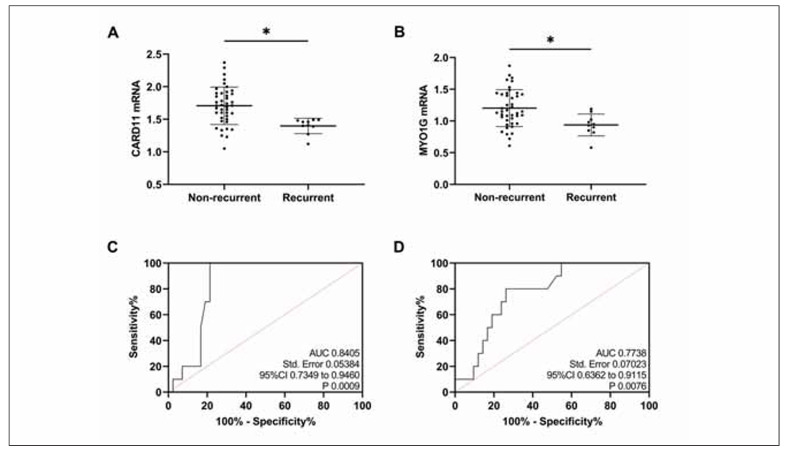
Assessment of prognosis of recurrence.<br>A: Comparison of CARD11. B: Comparison of MYO1G. C: ROC curve of prognostic recurrence of PTB diagnosed by CARD11. D: ROC curve of prognostic recurrence of PTB diagnosed by MYO1G. *P < 0.05.

Prognostic follow-up showed that PTB recurred in 10 patients, and CARD11 and MYO1G mRNA levels in relapsed patients were notably lower than those in non-relapsed patients (P<0.05, Figure 5A, Figure 5B). When CARD11 mRNA was <1.50, its sensitivity and specificity in diagnosing the prognosis of PTB recurrence were 100.00% and 78.57%, respectively (P<0.05, Figure 5C); when MYO1G mRNA was <1.03, its sensitivity and specificity in diagnosing prognosis of PTB recurrence were 80.00% and 73.81%, respectively (P<0.05, Figure 5D).

## Discussion

Gene function can be regulated at multiple levels, and the analysis of multi-omics data helps better understand the complex biological processes in human diseases [10]. In this study, we downloaded PTB expression profiling data from the GEO database, screened TB-related biomarkers, analysed the functional patterns with bioinformatics, and finally identified 52 genes differentially expressed between PTB patients and healthy controls. GO analysis provides a uniform standard language for describing the function of genes and gene products from different organisms, facilitating comparability across studies. Categorising genes and gene products into specific biological processes, cellular components, and molecular functional categories helps researchers understand the particular functions and biological processes of genes. KEGG analysis, on the other hand, can help researchers gain a deeper understanding of the role of genes in the complex network of organisms, which can reveal the intrinsic mechanisms of biological phenomena and provide new perspectives and clues for biological research [11]. In this study, we found through functional enrichment analysis that the differentially expressed genes mainly participate in biological processes of interferon signalling, immune defence response, cell apoptosis, and inflammatory response, primarily involving complement and coagulation cascades, the NOD-like receptor pathway, the cytokine-receptor interactions, and the TNF signalling pathways. The correlation of these pathways and functions with PTB has been wellestablished, further confirming the significant research potential of these DEGs in PTB.

T cells are one of the most important immune cells in the human body and an important component of the immune system [12]. They can regulate the body’s immune function by producing and releasing various cytokines, thus playing a particular role in the resistance to PTB; for the diagnosis of PTB, the T cell spot test for PTB infection is an essential auxiliary means of assessment [13]. T cells have a significant association with the occurrence and progression of PTB, which has been verified many times in previous studies [14] [15]. In the present study, we found that CARD11 and MYO1G in these DEGs were closely related to T cell function, which piqued our interest. CARD11 is an important signalling molecule that plays various roles in the immune system and other cells, primarily T lymphocytes, B lymphocytes, and dendritic cells [16]. In previous studies, CARD11 has been used to modulate T cell activation to improve the drug resistance of tumour cells [17]. However, its relationship with PTB has not been fully understood. MYO1G is a hemopoietic specific myosin that is localised at the plasma membrane and regulates cellular elasticity. Previous studies mainly focused on its use in tumour diseases [18] [19].

Thus, after the inclusion of clinical cases, it was found that CARD11 and MYO1G were lower in the research group than in the control group, which was consistent with the results of GEO2R analysis, suggesting that both of them may be involved in the occurrence and progression of PTB. Similarly, in melanoma, lung cancer, and other diseases, we found that both CARD11 and MYO1G were underexpressed [20] [21]. Subsequently, the ROC curve analysis revealed that CARD11 and MYO1G were excellent in diagnosing PTB occurrence, indicating their specific diagnostic role. In addition, the prognostic follow-up showed that these two were closely related to PTB recurrence and exhibited excellent effects in prognosis assessment. These results hold significant implications for future clinical evaluation of PTB condition. At present, the diagnosis and treatment of PTB in clinical practice mainly rely on the results of bacterial culture that requires high-quality samples (generally bronchoalveolar lavage fluid) and lacks timeliness of detection as bacterial culture and drug-sensitive test, takes about 7 days, which, not only unfavourable to the dynamic clinical assessment of PTB but also makes it difficult to achieve early predictive intervention, thus increasing the risk of poor prognosis. In contrast, the testing for molecular markers such as CARD11 and MYO1G can be directly performed through blood markers. The objective and quantitative test methods eliminate the risks of missed diagnosis or misdiagnosis due to human factors, which is significant for the clinical diagnosis and treatment of PTB.

Of course, the clinical application of these molecular markers still requires extensive clinical trials for validation. For instance, we need to determine whether changes in CARD11 and MYO1G promote the progression of PTB or if the progression of PTB leads to alterations in the expression of these genes. Additionally, the pathways and mechanisms of how CARD11 and MYO1G affect PTB should be figured out. In the future, we will conduct more comprehensive supplementary experiments to address these limitations and provide more reliable and extensive references and guidance for clinical practice.

## Conclusion

Through an online database, CARD11 and MYO1G were found to be DEGs closely related to T cell activation in PTB. Further clinical case studies revealed that CARD11 and MYO1G showed low expression in PTB and excellent effects in assessing PTB occurrence and prognosis of recurrence. These findings suggest that CARD11 and MYO1G can potentially be future PTB condition assessment indicators.

## Dodatak

### Availability of data and materials

The data used to support the findings of this study are available from the corresponding author upon request.

### Funding

Not applicable.

### Authors’ contribution

Fengxia Liu and Shengxun Lin contributed equally to this work and are co-first authors.

### Conflict of interest statement

All the authors declare that they have no conflict of interest in this work.
